# An in vitro evaluation of marginal fit zirconia crowns fabricated by a CAD-CAM dental laboratory and a milling center

**DOI:** 10.1186/s12903-019-0810-9

**Published:** 2019-06-13

**Authors:** Avi Meirowitz, Yoli Bitterman, Sharon Levy, Eitan Mijiritsky, Eran Dolev

**Affiliations:** 1 Department of Oral Rehabilitation, Goldschleger School of Dental Medicine, The Sackler Faculty of Medicine, Tel Aviv University, Tel Aviv, Israel; 2 Department of Orthodontics, Goldschleger School of Dental Medicine, The Sackler Faculty of Medicine, Tel Aviv University, 69978 Tel – Aviv, Israel; 3 Sackler Faculty of Medicine, Department of Otolaryngology Head and Neck and Maxillofacial Surgery, Tel-Aviv Sourasky Medical Center, Tel-Aviv University, Tel Aviv, Israel

**Keywords:** CAD-CAM, Zirconia, Crown, Marginal fit, CEREC, LAVA

## Abstract

**Background:**

Marginal fit is critical for the success and longevity of a dental restoration. Zirconia crowns can be fabricated either chair-side, in a dental laboratory or in a milling center; each can give different marginal fits results. However, discussion of the marginal fit of zirconia crowns when different fabrication methods are compared is lacking in the literature.

**Purpose:**

To compare the marginal discrepancy (MD) and absolute marginal discrepancy (AMD) of computer-aided design, and computer-aided manufacturing (CAD-CAM) used in a dental laboratory and a milling center for producing monolithic zirconia crowns.

**Methods:**

The marginal fit of 30 zirconia crowns cemented to typodont teeth was evaluated by means of a sectioning technique. Fifteen crowns were fabricated with a CEREC inLAB MC X5 from IPS e.max ZirCAD blocks. Fifteen crowns were fabricated using a LAVA milling center from LAVA Plus Zirconia Blocks. The 30 crowns were sectioned with a precision saw, and MD and AMD were subsequently measured using a light microscope. Data were analyzed using the one-way ANOVA technique to investigate significant differences in the marginal fit between the two fabrication systems (α = .05).

**Results:**

The AMD dimension of the CEREC inLAB system was significantly smaller (*P* < .05). Mean AMD values for zirconia crowns fabricated by the CEREC inLAB were 85 μm, and for the LAVA milling center 133 μm. There was no significant difference between the two systems regarding the MD dimensions. The MD values for zirconia crowns fabricated by the CEREC inLAB were 53 μm and for the LAVA milling center 61 μm.

**Conclusions:**

The CEREC inLAB system demonstrated significantly better marginal fit in relation to the AMD. However, no difference between the systems was found in the MD. Monolithic zirconia crowns fabricated by the CAD-CAM CEREC inLAB system and the LAVA system milling center showed MD values of less than 120 μm, which is within the clinically acceptable range.

## Background

In recent decades, increasing demand from patients for natural-appearing dental restorations has led to the development of all-ceramic materials with improved mechanical characteristics that ensure suitable longevity. These are now replacing traditional metal-ceramic restorations [1–3]. The introduction of CAD-CAM technology allows for the use of materials such as zirconia, which is free of metal, in dental restorations [1].

Zirconia is a polycrystalline ceramic without a glassy phase and exists in several temperature-dependent forms. At room temperature, it exists in a monoclinic crystalline form, changing to a tetragonal and cubic crystalline form when sintered [4]. The cooling from cubic to tetragonal results in an expansion of 2.3% and from tetragonal to monoclinic of 4.2%. These expansions are the cause of cracks and hence there is a need to stabilize the tetragonal form. The most common method of stabilizing the tetragonal phase and maintaining zirconia in a metastable condition at room temperature is achieved via the addition of a small amount of yttria to the zirconia [3–5]. Such treatment produces a stronger material than other available ceramics. The zirconia is a biocompatible material with high mechanical properties of 1200 HV hardness, 900–1200 MPa flexural strength and fracture toughness of 6–8 MPa m^1/2^ [6, 7].

Zirconia restorations fabricated by CAD-CAM technology can be produced chair-side, in a laboratory or in a milling center. The restorations are processed either by soft machining of pre-sintered blanks with enlarged contours followed by sintering at high temperature during which they shrink to their desired and final size, or by hard machining of fully sintered blocks [8].

Superior marginal fit is an important characteristic for the success and longevity of dental restorations. Poor marginal fit results in plaque retention and microleakage; this can lead to secondary dental caries, pulpal lesions, periodontal disease, and bone loss [9, 10]. Although dental literature includes significant investigation of the accuracy of marginal fit, there is no consensus on the maximum acceptable marginal discrepancy. Marginal discrepancies of between 50 and 120 μm are considered clinically acceptable as regards longevity of the restoration, while more restrictive studies proposed marginal discrepancies of less than 100 μm [11, 12]. An in vivo study of more than 1000 crowns found a greater association between a marginal discrepancy of less than 120 μm and higher longevity [13]. Studies on the marginal fit of zirconia copings fabricated by CAD-CAM have reported measured marginal discrepancies of as low as 10 μm and as high as 160 μm, with most being less than 80 μm [14–16]. With regard to full zirconia crown, the studies show marginal discrepancies between 11 μm to 58 μm [17, 18].

**Fig. 1:**
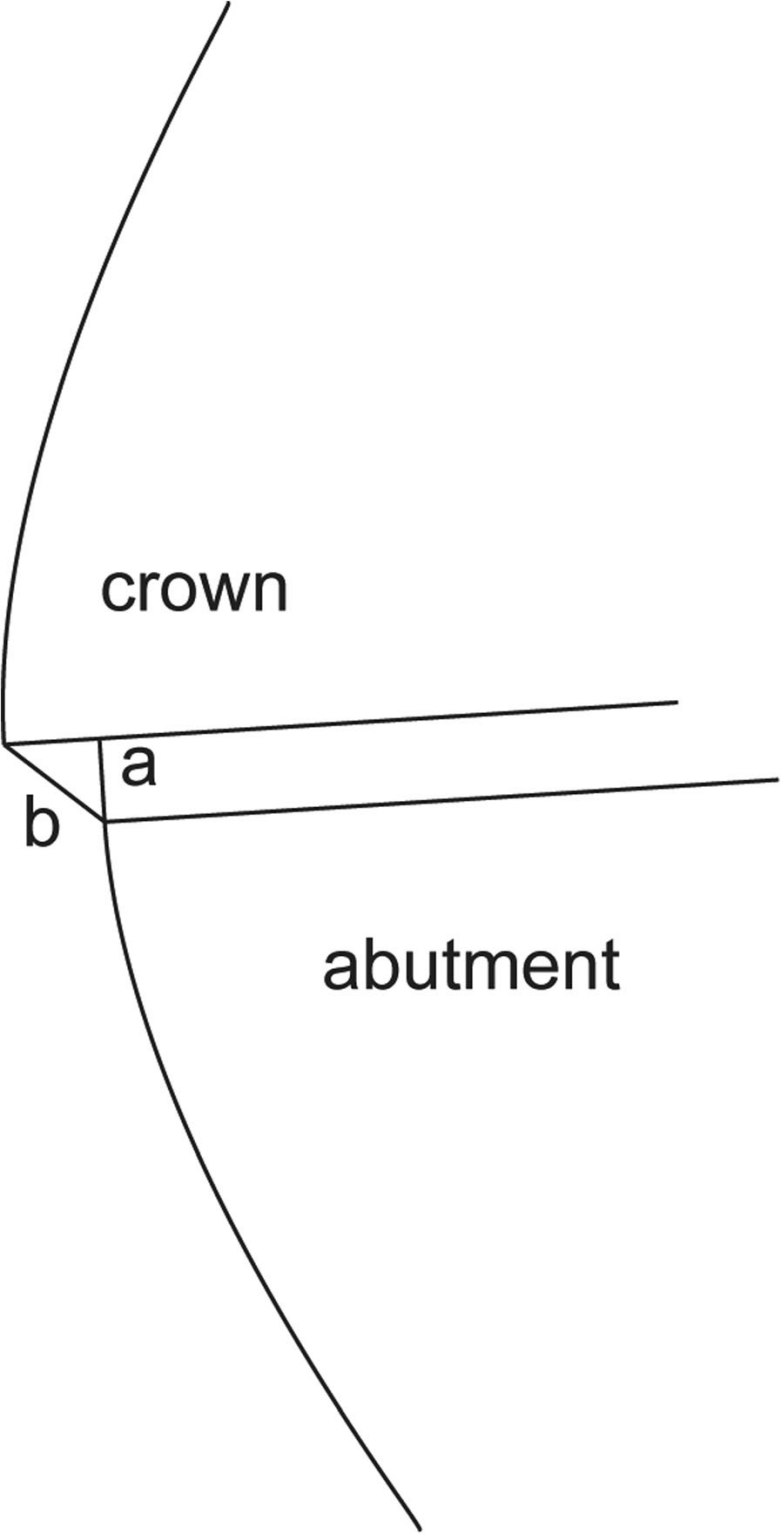
Discrepancies between crown and abutment finish line. **a** Marginal discrepancy (MD); **b** Absolute marginal discrepancy (AMD) [25]

The definition of marginal fit can differ and depends on the gap measured in the studies. Holmes et al. defined the marginal discrepancy (MD) as the perpendicular measurement from the cervical margin of the restoration to the preparation margin, while the AMD is measured from the cervical margin of the restoration to the cavosurface of the preparation [19]. The MD represents the surface of the cement that is exposed to the oral environment and can be dissolved, resulting in microleakage. The AMD is indicative of the under- or over-extension of the restoration margins relative to the margins of the preparation and plays a significant role in plaque accumulation (Fig. 1) [19].

Studies that have compared the marginal fit of zirconia copings to other ceramic restorations show higher accuracy for zirconia [20]. The marginal fit of zirconia copings produced using different CAD-CAM system has also been investigated [15, 16, 21, 22]. The marginal fit of monolithic zirconia crown was studied with regard to different preparation designs and sintering techniques [17, 18]. However, no studies investigate the effect of different CAD-CAM fabrication methods on the marginal fit of monolithic zirconia crowns; the use of monolithic zirconia crowns is increasing, and therefore a comparison of fabrication methods is justified. The purpose of this in vitro study was thus to compare the marginal fit of monolithic zirconia crowns produced using two fabrication methods: dental laboratory and milling center. The null hypothesis was that no difference would be found in the marginal fit of the fabrication methods.

Many methods exist for the evaluation of marginal fit using non-disruptive methods like silicone paste technique [12], micro-CT scan [20, 23], and disruptive methods, which include sectioning with a disk [21, 24]. In this in vitro study, the sectioning method was used on typodont teeth to investigate the two parameters of marginal fit, AMD and MD.

## Material and methods

**Fig. 2:**
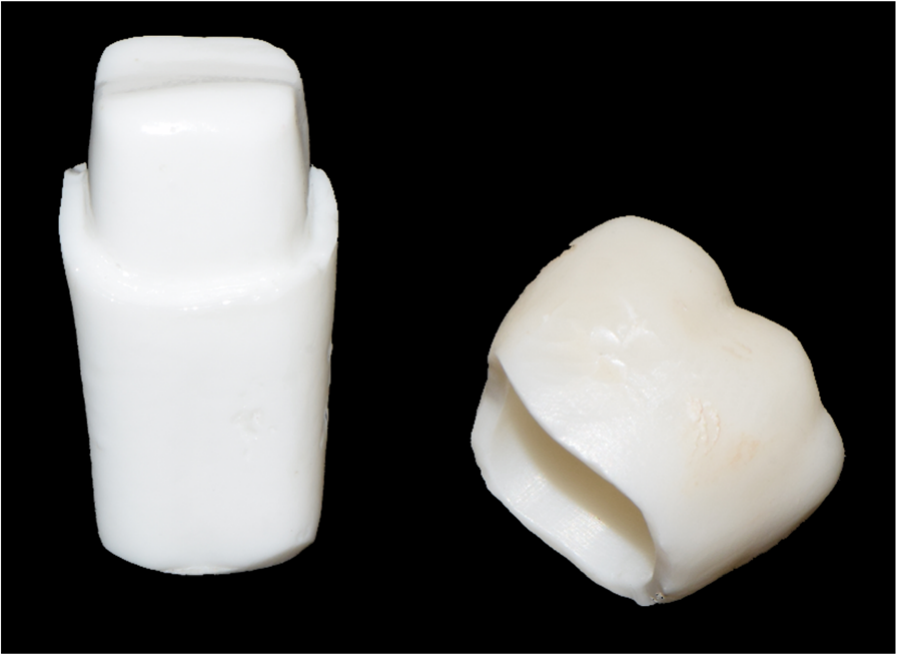
Lower left first molar typodont tooth and corresponding CEREC inLAB zirconia crown

The following method is the same as that previously published by Dolev et al. and will be described here only briefly [25]. Mandibular left first molar, typodont teeth (FLUX 8634; Columbia Dentoform) were used as abutment. For the CEREC inLAB system group, 15 typodont teeth were scanned with an intraoral scanner (CEREC SW 4.52, CEREC Omnicam scanner; Dentsply Sirona) by dentists with experience using CAD-CAM systems, who also marked the finish line using CAD system (CEREC Connect SW 4.1 software; Dentsply Sirona). The 15 crowns were prepared in a dental laboratory (TOTALI - AMIR LIFF LTD, Tel Aviv, Israel) by master dental technician (MDT). They were formed from partially sintered zirconia blocks (IPS e.max ZirCAD; Ivoclar Vivadent) using a CAM milling unit (CEREC inLAB MC X5; Dentsply Sirona), followed by sintering (Ceramill Therm 1; Amann Girrbach) to produce completely sintered crowns (Fig. 2). The CAD-CAM parameters were as followed: Spacer (radial) – 90 μm, Spacer occlusal – 100 μm, Proximal contacts strength – 25 μm, Minimal thickness (radial) – 700 μm, Minimal thickness (occlusal) – 1500 μm, Marginal thickness – 50 μm, Marginal ramp width – 150 μm, Marginal ramp angle – 45°. Fifteen typodont teeth were sent to a milling center (LAVA; 3 M ESPE) for scanning (Lava Scan ST; 3 M ESPE) and design (LAVA Design 5; 3 M ESPE). The zirconia crowns were fabricated by partially sintered zirconia blocks (LAVA Plus Zirconia Blocks; 3 M ESPE) using a CAM milling machine (LAVA Form; 3 M ESPE) followed by sintering (LAVA Furnace 200; 3 M ESPE) for production of the final crowns. The parameters of both the CAD-CAM systems were identical.

**Fig. 3:**
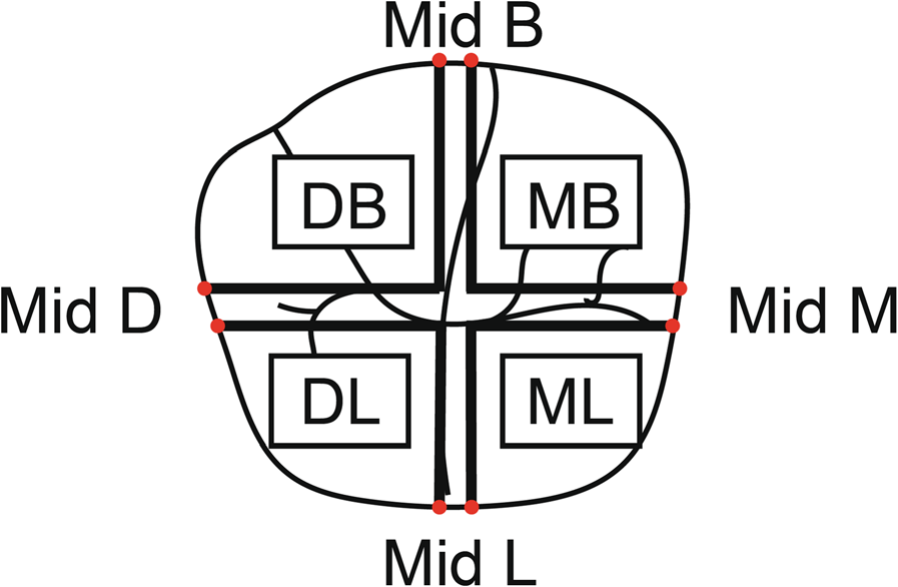
Illustration of sectioned mandibular left first molar. Colored points demonstrate eight measurement locations of finish line discrepancies

The crowns were cemented with self-adhesive resin cement (Rely X U-200; 3 M ESPE) and then sectioned with a cutting machine (Izomet Plus precision saw; Buehler), creating four specimens from each crown: Mesio-Buccal (MB), Disto-Buccal (DB), Disto-Lingual (DL), and Mesio-Lingual (ML). In each specimen, the AMD and MD were measured in two locations (Fig. 3) using a light microscope (Axioplan 2; Zeiss) at × 110 magnification [21].

Repeat measurements using the one-way ANOVA statistical test were carried out (α = .05) to examine significant differences between the groups.

## Results

**Fig. 4:**
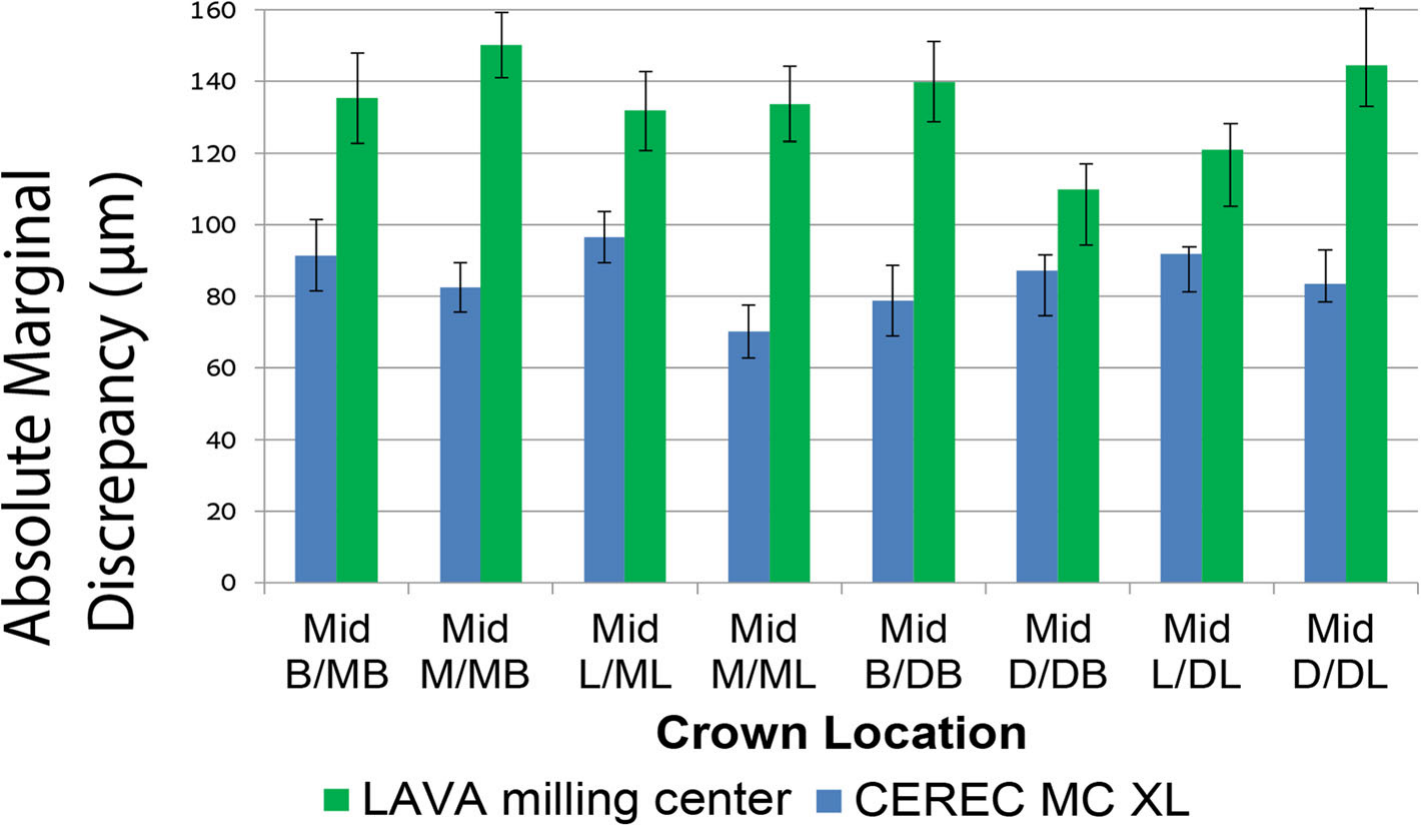
Comparison of mean values and standard errors of AMD at different marginal area locations for CEREC inLAB system and LAVA milling center crowns

**Fig. 5:**
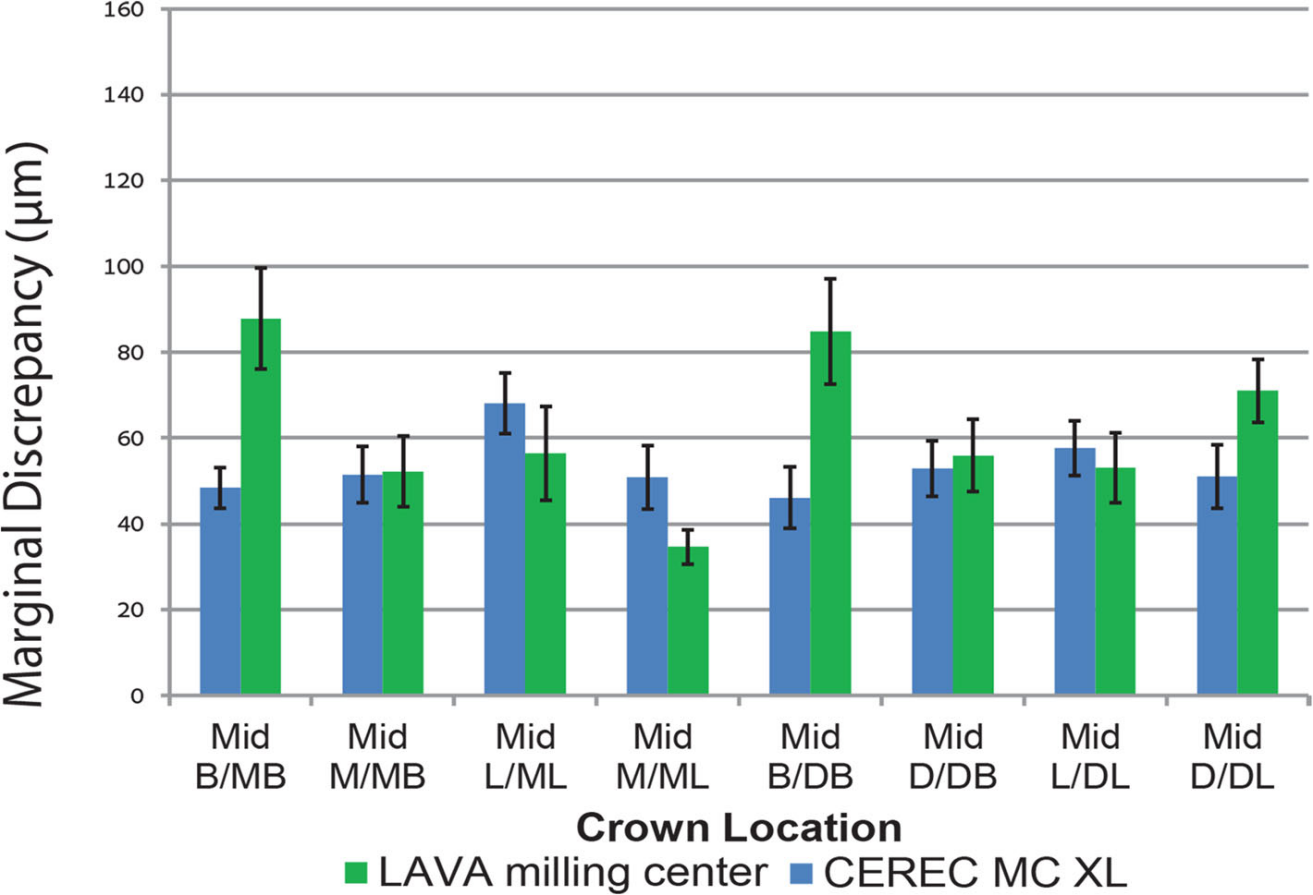
Comparison of mean values and standard errors of MD at different marginal area locations for CEREC inLAB system and LAVA milling center crowns

Figures 4 and 5 show the mean values with standard errors for the AMD and MD dimensions as measured in 8 locations for crowns fabricated by the CEREC inLAB and LAVA milling center. The CEREC inLAB presented smaller AMD values than the LAVA milling center (Fig. 4). The MD values of CEREC inLAB crowns were smaller than those produced in the LAVA milling center, except for Mid-L/ML, Mid-L/DL, and Mid-M/ML locations (Fig. 5).

The overall mean ± standard error (SE) value for AMD and MD of the CEREC inLAB and LAVA milling center fabrication methods are presented in Table 1. The statistical outcome showed significant differences for AMD (*df* = 1, F = 35.081; *P* = .000) whereas MD yielded no significant differences (*df* = 1, F = 1.799; *P* = .191) between CEREC inLAB and the LAVA milling center. The MD 95% confidence intervals are presented in Table 1.

**Table 1:** Overall mean ± standard error (SE) and 95% confidence intervals of the fabrication methods

	AMD mean ± SE	MD mean ± SE	MD 95% confidence intervals-Lower bound	MD 95% confidence intervals-upper bound
CEREC inLAB	85 ± 2 μm*	53 ± 2 μm	43 μm	62 μm
LAVA milling center	133 ± 4 μm*	61 ± 3 μm	52 μm	71 μm

*significant difference

## Discussion

In accordance with the study results, the null hypothesis regarding the AMD parameter was rejected, with the dental laboratory using the CEREC inLAB CAD-CAM displaying a significantly lower gap (85 ± 2 μm) compared to the LAVA milling center (133 ± 4 μm). For the MD parameter, the null hypothesis was not rejected since no statistically significant differences were found between the two systems.

Beuer et al. identified significant differences when examining the MD of 3-unit zirconia frameworks [21]. They found a smaller mean MD value in the milling center (29.1 μm) than in frameworks produced by CEREC inLAB (56.6 μm). However, this study made use of an Etkon milling center and a traditional silicone impression technique rather than an intraoral scanner. Studies that compared marginal fit of zirconia coping found that the CEREC inLAB system showed smaller MD values compared to other CAD-CAM systems [15, 16, 22]. Rajan et al. compared the marginal fit of zirconia coping produced by CEREC inLAB with that of the CERAMILL system and found significant differences, whereby CEREC inLAB copings had better adaptation than CERAMILL. For both CAD-CAM systems, a digital scanner was used [22]. Marginal fit for CERAMILL was 83 μm and for the CEREC InLAB MC XL was 68 μm [22]. Saab et al. compared marginal fit of zirconia coping with four different CAD-CAM systems: CEREC inLAB, CERCON, CERAMILL, and LAVA milling unit. They used a specific intraoral scanning device for each of the CAD-CAM systems. CEREC inLAB showed significant lower mean value of MD, 37.68 μm [16]. ArRejaie et al. compared marginal fit of zirconia coping with 3 different CAD-CAM systems: DeguDent, KaVo Everest, and Lava Ultimate. They also digitized their model with an intraoral scanner of the specific CAD-CAM unit. Lava Ultimate showed a significantly lower MD mean value of 112.5, (statistically significant compared to KaVo Everest) [15], this MD value is relatively high compared to previous mentioned studies. Another study by Beuer et al. examined the marginal gap of 3-unit zirconia framework using two different fabrication concepts. One was fabricated by a laboratory system (Cercon Brain, DeguDent) and the other in a milling center (Compartis Integrated Systems, DeguDent). In their study, both fabrication systems used a polyether impression technique, the same CAD-CAM system and porous zirconia, but a different milling unit. They found no significant differences between the two fabrication methods regarding the marginal gap [26].

In their systematic review of the fit of zirconia restorations, Abduo et al. [27]. indicated the difficulty in comparing the many studies existing on the marginal gap of zirconia given the different methodology used in each study [27], including the sectioning technique [17, 20], use of microcomputed tomography [20, 23], and silicone paste technique [12]. Additionally, each study examined and compared different parameters of marginal fit, MD and AMD being a few of the many that were described by Holmes et al. [19]. Hence there is a need for standardization.

Crown cementation has been used in the present study to reproduce the clinical conditions of the crown-abutment relationship. Earlier publications have found that crown cementation has a negative effect on the marginal fit, which increases after cementation [23, 28].

McLean and Fraunhofer showed that crown marginal discrepancies ranging up to 120 μm were clinically acceptable [13]. According to the 95% confidence interval, the present study yielded MD values within the clinically acceptable range.

This study examined MD and AMD in four surfaces: buccal, lingual, mesial, and distal. It did not compare those parameters between the different surfaces because this is clinically irrelevant given that this study used model teeth with a constant finish line, in an in-vitro setting.

Several limitations were identified in the study, as follows: The study was conducted in vitro with typodont teeth used as abutments instead of natural teeth, and finger pressure was used to lute the crowns. These characteristics differ from those of the intraoral environment. The cemented crowns were cut with a disk, a destructive method, which can have negative effects on the quality of specimens and the reading of marginal fit. Additionally, the cement thickness in the occlusal area that affects the internal marginal fit and the seating quality of the crowns was not measured, and could therefore have influenced the MD. One other limitation is the fact that the two CAD-CAM systems used zirconia blocks manufactured by different companies, which may also have affected the results.

The study revealed that when using a well-known, established CAD-CAM system, zirconia monolithic crowns are a good treatment option as regards marginal fit during tooth restoration. This is of relevance given the popularity of zirconia monolithic crowns as a treatment option. Because these systems are constantly developing with the arrival of new manufacturers, further in vitro and in vivo studies are needed to substantiate these results.

## Conclusions

Within the limitations of this study, the following conclusions were drawn:

1. The CEREC inLAB system shows a significantly smaller AMD than the LAVA milling center.
2. No significant difference was found in MD between the systems.
3. Monolithic zirconia crowns fabricated by the CEREC inLAB system and the LAVA milling center produced MD values within the clinically acceptable standard (120 μm).
4. There is a need for standard rules and guidance when comparing marginal fit between different CAD-CAM systems.

## Data Availability

The datasets used and/or analysed during the current study are available from the corresponding author upon reasonable request.

## Abbreviations

AMD: Absolute marginal discrepancy
CAD-CAM: computer-aided design and computer-aided manufacturing
DB: Disto Buccal
DL: Disto Lingual
MB: Mesio Buccal
MD: Marginal discrepancy
ML: Mesio Lingual
SE: Standard error

## References

[1] Raigrodski AJ, Hillstead MB, Meng GK, Chung KH (2012). Survival and complications of zirconia-based fixed dental prosthesis: a systematic review. J Prosthet Dent.

[2] Biscaro L, Bonfiglioli R, Soattin M, Vigolo P (2013). An in vivo evaluation of fit of zirconium-oxide based ceramic single crowns, generated with two CAD/CAM systems, in comparison to metal ceramic single crowns. J Prosthodont.

[3] Zarone F, Russo S, Sorrentino R (2011). From porcelain-fused-to-metal to zirconia: clinical and experimental considerations. Dent Mater.

[4] Denry I, Kelly JR (2008). State of the art of zirconia for dental applications. Dent Mater.

[5] Christel P, Meunier A, Heller M, Torre JP, Peille CN (1989). Mechanical properties and short-term in-vivo evaluation of yttrium-oxide-partially-stabilized zirconia. J Biomed Mater Res.

[6] Piconi C, Maccauro G (1999). Zirconia as a ceramic biomaterial. Biomaterials.

[7] Kosmac T, Oblak C, Jevnikar P, Funduk N, Marion L (1999). The effect of surface grinding and sandblasting on flexural strength and reliability of Y-TZP zirconia ceramic. Dent Mater.

[8] Filser F, Kocher P, Gauckler LJ (2003). Net-shaping of ceramic components by direct ceramic machining. Assembly Autom.

[9] Sorensen JA (1989). A rationale for comparison of plaque-retaining properties of crown systems. J Prosthet Dent.

[10] Kashani HG, Khera SC, Gulker IA (1981). The effects of bevel angulation on marginal integrity. J Am Dent Assoc.

[11] Suárez MJ, González de Villaumbrosia P, Pradíes G, Lozano JF (2003). Comparison of the marginal fit of procera AllCeram crowns with two finish lines. Int J Prosthodont.

[12] Coli P, Karlsson S (2004). Fit of a new pressure-sintered zirconium dioxide coping. Int J Prosthodont.

[13] McLean JW, von Fraunhofer JA (1971). The estimation of cement film thickness by an in vivo technique. Br Dent J.

[14] Boitelle P, Mawussi B, Tapie L, Fromentin O (2014). A systematic review of CAD/CAM fit restoration evaluations. J Oral Rehabil.

[15] ArRejaie A, Alalawi H, Al-Harbi FA, Abualsaud R, Al-Thobity AM (2018). Internal fit and marginal gap evaluation of zirconia copings using microcomputed tomography: an in vitro analysis. Int J Periodontics Restorative Dent.

[16] Saab RC, da Cunha LF, Gonzaga CC, Mushashe AM, Correr GM (2018). Micro-CT analysis of Y-TZP copings made by different CAD/CAM Systems: marginal and internal fit. Int J Dent.

[17] Ahmed WM, Abdallah MN, McCullagh AP, Wyatt CCL, Troczynski T, Carvalho RM (2019). Marginal discrepancies of monolithic zirconia crowns: the influence of preparation designs and sintering techniques. J Prosthodont.

[18] Carbajal Mejía JB, Yatani H, Wakabayashi K, Nakamura T (2019). Marginal and internal fit of CAD/CAM crowns fabricated over reverse tapered preparations. J Prosthodont.

[19] Holmes JR, Bayne SC, Holland GA, Sulik WD (1989). Considerations in measurement of marginal fit. J Prosthet Dent.

[20] Riccitiello F, Amato M, Leone R, Spagnuolo G, Sorrentino R (2018). In vitro evaluation of the marginal fit and internal adaptation of zirconia and Lithium Disilicate single crowns: micro-CT comparison between different manufacturing procedures. Open Dent J.

[21] Beuer F, Aggstaller H, Edelhoff D, Gernet W, Sorensen J (2009). Marginal and internal fits of fixed dental prostheses zirconia retainers. Dent Mater J.

[22] Rajan BN, Jayaraman S, Kandhasamy B, Rajakumaran I (2015). Evaluation of marginal fit and internal adaptation of zirconia copings fabricated by two CAD-CAM systems: an in vitro study. The Journal of the Indian Prosthodontic Society.

[23] Demir N, Ozturk AN, Malkoc MA (2014). Evaluation of the marginal fit of full ceramic crowns by the microcomputed tomography (micro-CT) technique. Eur J Dent.

[24] Sulaiman F, Chai J, Jameson LM, Wozniak WT (1997). A comparison of the marginal fit of in-Ceram, IPS empress, and procera crowns. Int J Prosthodont.

[25] Dolev E, Bitterman Y, Meirowitz A (2019). Comparison of marginal fit between CAD-CAM and hot-press lithium disilicate crowns. J Prosthet Dent.

[26] Beuer F, Korczynski N, Rezac A, Naumann M, Gernet W, Sorensen JA (2010). Marginal and internal fit of zirconia based fixed dental prostheses fabricated with different concepts. Clin Cosmet Investig Dent.

[27] Abduo J, Lyons K, Swain M (2010). Fit of zirconia fixed partial denture: a systematic review. J Oral Rehabil.

[28] Borges GA, Faria JS, Agarwal P, Spohr AM, Correr-Sobrinho L, Miranzi BA (2012). In vitro marginal fit of three all-ceramic crown systems before and after cementation. Oper Dent.

